# Supplementary material for: Machine Learning Approaches for the Prioritization of Genomic Variants Impacting Pre-mRNA Splicing
Source: Cells. 2019 Nov 26;8(12):1513. doi: 10.3390/cells8121513 (PMC6953098; doi:10.3390/cells8121513)
Supplement: Supplementary file 1 [file cells-08-01513-s001.pdf]

Supplementary Materials

Table S1. Summary of bioinformatics tools with data access details. Tabulated list of 7 bioinformatics tools with links to online data access (links correct and functional as of 15 November 2019). PMID, PubMed ID.

Tool	Purpose	Link	PMID	Citation
CADD	General-purpose pathogenicity scoring	Tabulated datasets: https://cadd.gs.washington.edu/download Online lookup: https://cadd.gs.washington.edu/snv	30371827, 24487276	[1,2]
TraP	Quantification of variant impact on transcripts	Request for access to tabulated scores: http://innovation.columbia.edu/technologies/cu17233_pathogenicity-database-for-identification-of-disease-causing-non-coding-genetic-variations Online lookup: http://trap-score.org/Search?version=v3	28794409	[3]
SPANR	Cassette exon skipping prediction	Online lookup: http://tools.genes.toronto.edu Link for ANNOVAR download: http://annovar.openbioinformatics.org/en/latest/user-guide/download/	25525159	[4]
CryptSplice	Effect of variants on existing splice sites and cryptic splice site prediction	Bitbucket source code download: https://bitbucket.org/jhucidr/cryptsplice/src/master/	28475858	[5]
MMSplice	Prediction of exon skipping, competitive interactions, changes in splicing efficiency and pathogenicity	GitHub source code download: https://github.com/gagneurlab/MMSplice	30823901	[6]
S-CAP	Variant pathogenicity scoring with the compartmentalization of genomic space	Tabulated datasets: http://bejerano.stanford.edu/scap/ Bitbucket source code download: https://bitbucket.org/bejerano/splicing_classifier/src/master/	30804562	[7]
SpliceAI	Prediction of variant impact on acceptor/donor loss or gain	GitHub source code download: https://github.com/Illumina/SpliceAI	30661751	[8]

Reference

1. Rentzsch, P.; Witten, D.; Cooper, G.M.; Shendure, J.; Kircher, M. CADD: Predicting the deleteriousness of variants throughout the human genome. *Nucleic Acids Res.* **2019**, *47*, D886-D894, doi:10.1093/nar/gky1016.
2. Kircher, M.; Witten, D.M.; Jain, P.; O'Roak, B.J.; Cooper, G.M.; Shendure, J. A general framework for estimating the relative pathogenicity of human genetic variants. *Nat. Genet.* **2014**, *46*, 310–315, doi:10.1038/ng.2892.
3. Gelfman, S.; Wang, Q.; McSweeney, K.M.; Ren, Z.; La Carpia, F.; Halvorsen, M.; Schoch, K.; Ratzon, F.; Heinzen, E.L.; Boland, M.J.; et al. Annotating pathogenic non-coding variants in genic regions. *Nat. Commun.* **2017**, *8*, 236, doi:10.1038/s41467-017-00141-2.
4. Xiong, H.Y.; Alipanahi, B.; Lee, L.J.; Bretschneider, H.; Merico, D.; Yuen, R.K.; Hua, Y.; Gueroussov, S.; Najafabadi, H.S.; Hughes, T.R.; et al. RNA splicing. The human splicing code reveals new insights into the genetic determinants of disease. *Science* **2015**, *347*, 1254806, doi:10.1126/science.1254806.
5. Lee, M.; Roos, P.; Sharma, N.; Atalar, M.; Evans, T.A.; Pellicore, M.J.; Davis, E.; Lam, A.N.; Stanley, S.E.; Khalil, S.E.; et al. Systematic Computational Identification of Variants That Activate Exonic and Intronic Cryptic Splice Sites. *Am. J. Hum. Genet.* **2017**, *100*, 751–765, doi:10.1016/j.ajhg.2017.04.001.

6. Cheng, J.; Nguyen, T.Y.D.; Cygan, K.J.; Çelik, M.H.; Fairbrother, W.G.; Avsec, Ž.; Gagneur, J. MMSplice: Modular modeling improves the predictions of genetic variant effects on splicing. *Genome Biol.* **2019**, *20*, 48, doi:10.1186/s13059-019-1653-z.
7. Jagadeesh, K.A.; Paggi, J.M.; Ye, J.S.; Stenson, P.D.; Cooper, D.N.; Bernstein, J.A.; Bejerano, G. S-CAP extends pathogenicity prediction to genetic variants that affect RNA splicing. *Nat. Genet.* **2019**, *51*, 755–763, doi:10.1038/s41588-019-0348-4.
8. Jaganathan, K.; Kyriazopoulou Panagiotopoulou, S.; McRae, J.F.; Darbandi, S.F.; Knowles, D.; Li, Y.I.; Kosmicki, J.A.; Arbelaez, J.; Cui, W.; Schwartz, G.B.; et al. Predicting Splicing from Primary Sequence with Deep Learning. *Cell* **2019**, *176*, 535–548, doi:10.1016/j.cell.2018.12.015.
